# Modulation of proper name recall by transcranial direct current stimulation of the anterior temporal lobes

**DOI:** 10.1038/s41598-022-09781-x

**Published:** 2022-04-06

**Authors:** Shane Fresnoza, Rosa-Maria Mayer, Katharina Sophia Schneider, Monica Christova, Eugen Gallasch, Anja Ischebeck

**Affiliations:** 1 Institute of Psychology, University of Graz, Universitätsplatz 2/DG, 8010 Graz, Austria; 2 Section of Physiology, Otto Loewi Research Center, Medical University of Graz, Graz, Austria; 3 BioTechMed, Graz, Austria; 4 Institute for Physiotherapy, University of Applied Sciences, FH-Joanneum, Graz, Austria

**Keywords:** Neuroscience, Physiology, Psychology

## Abstract

We often fail to recall another person's name. Proper names might be more difficult to memorize and retrieve than other pieces of knowledge, such as one's profession because they are processed differently in the brain. Neuroimaging and neuropsychological studies associate the bilateral anterior temporal lobes (ATL) in the retrieval of proper names and other person-related knowledge. Specifically, recalling a person's name is thought to be supported by the left ATL, whereas recalling specific information such as a person's occupation is suggested to be subserved by the right ATL. To clarify and further explore the causal relationship between both ATLs and proper name retrieval, we stimulated these regions with anodal, cathodal and sham transcranial direct current stimulation (tDCS) while the participants memorized surnames (e.g., Mr. Baker) and professions (e.g., baker) presented with a person’s face. The participants were then later asked to recall the surname and the profession. Left ATL anodal stimulation resulted in higher intrusion errors for surnames than sham, whereas right ATL anodal stimulation resulted in higher overall intrusion errors, both, surnames and professions, compared to cathodal stimulation. Cathodal stimulation of the left and right ATL had no significant effect on surname and profession recall. The results indicate that the left ATL plays a role in recalling proper names. On the other hand, the specific role of the right ATL remaines to be explored.

## Introduction

Forgetting proper names such as a person's name can create an embarrassing situation that most of us want to avoid. The problems of recalling names can range from a "tip-of-the-tongue" state, that is the feeling that we have the target word just on the tip of our tongue but cannot recall it, to proper names anomia in neurological patients^1,2^. Proper names are assumed to be susceptible to forgetting because they refer to unique independent entities (e.g., specific persons, places, buildings, or brands) and thus lack the meaning in the sense in which common nouns have meanings (denoting categories of objects) or synonyms^1,3–5^. For instance, a person’s name is difficult to remember compared to conceptual biographical information describing that person, such as their profession^6^. This difficulty persists even in the case of name-profession homophones; a phenomenon called the "Baker-baker paradox"^2,3,5,7^. In other words, it is much harder to recall that a person's surname is Baker than to recall that a person is a baker.

Studies suggest that when "baker" is used as a common name, it infers probable characteristics of a person (e.g., “makes bread and cakes”, “sells bread and cakes”) and other attributes that are typical, even definitional, of this professional category^6^. This indicates that common names have a more detailed representation in semantic memory. In contrast, when “baker” is used as a proper name “Mr. Baker”, it conveys little information (e.g., he bears a common Anglo-Saxon name) about the person^6^. Moreover, common names are assumed to be linked directly to lexical nodes (via a visual and propositional node), whereas proper names are not directly connected to a lexical node because of an intermediate stage that mediates between conceptual and lexical information called the “proper noun phrase” or a “person identity node (PIN)”^6,8,9^. These predictions also fit with the framework of the earlier Bruce and Young model (1986) of face recognition. According to their model, face presentation evokes the construction of a visual percept of the face, which is then compared with the representations of faces stored in face recognition units (FRUs). Subsequently, the PINs containing semantic information about that person are accessed and this makes it possible to access the name code (the lexical unit corresponding to that person’s name)^10^. In other words, semantic information about the person (e.g., occupation, interest, etc.) can be easily accessed because they are contained in PINs that have direct bidirectional connections to the FRU (considered being the mental “lexicon” for faces), whereas names can just be accessed through PINs because they are not directly linked to the FRU^5,11,12^.

The neuroanatomical basis of proper name processing is not yet fully determined. Studies implicate the left brain hemisphere for the lexical access to proper names because of its dominance for language processing^1,13^. Normal retrieval of words that denote concrete entities (e.g., familiar persons), however, depends not just on classical Broca and Wernicke language areas, but also on regions in higher-order association cortices such the left anterior temporal lobe (left ATL)^14,15^. It is assumed that activation of the left ATL promotes the retrieval of lexical knowledge required for word production. Focal brain lesions in this region in patients with left hemisphere language dominance specifically impaired naming of famous person’s faces (e.g., "George Clooney") and famous landmarks (e.g., "Golden Gate Bridge")^14–18^. Several functional magnetic resonance imaging (fMRI) studies in healthy individuals also demonstrated an increase in left ATL activation, as well as increased functional connectivity between hippocampal and left ATL activation during the recall of familiar or famous and newly learned people's names^19–23^. Positron-emission tomography (PET) studies also reported left ATL activation when retrieving the names of unique persons and landmarks, as well as for famous faces relative to famous proper names and famous proper names relative to common names^14,24,25^. Recently, intracerebral recordings in epileptic patients showed that face and name identity were integrated in the left ventral ATL^26^.

The right ATL is also thought to contribute to the process of naming because bilateral ATL activations were observed in several PET and fMRI studies when naming familiar persons and during the categorization of persons by occupation^14,27,28^. Several patient studies suggest a distinct contribution of the right and left ATL. For instance, patients with unilateral epileptic foci in the right temporal pole were shown to have deficits in face recognition, semantic memory and naming, while patients with unilateral epileptic foci in the left temporal pole only had deficits in face naming^10,29^. Furthermore, patients with language-dominant temporal lobectomy showed an impaired ability to retrieve familiar people’s names, whereas patients with language-nondominant temporal lobectomy had difficulty associating newly-learned faces and names^10,20^. These results collectively suggest that right ATL damage predisposes a person to loss of familiarity (recognition deficits) and person-specific semantic information, whereas left ATL damage results in a prevalent difficulty in retrieving proper names (naming deficits)^6,30^. However, the dissociation is not absolute since other studies suggest that both anterior temporal lobes are necessary to access names^10^. In addition, fMRI studies show an extended network involved in retrieving person-specific information, including a visual processing core system formed by the inferior occipital areas and the fusiform face area assumed to code invariant facial features. Further activations include the posterior superior temporal sulcus, the temporo-parietal junction, the precuneus and left prefrontal and cingulate cortices^10,15,20,31^.

In recent years, there has been an increased interest in using non-invasive brain stimulation (NIBS) techniques such as transcranial direct current stimulation (tDCS) to further understand the neuropsychological underpinnings of proper name recall. In tDCS, a weak direct electrical current is applied to the scalp to transiently alter neuronal membrane potentials in the cortex underneath the electrodes. The effect of tDCS on neuronal activity was shown to be polarity-dependent because anodal and cathodal stimulation increase and decrease cortical excitability (as indexed by motor evoked potentials or MEPs), respectively^32^. Application of anodal and cathodal stimulation can therefore facilitate or inhibit cognitive processes, including proper name retrieval. Using tDCS can thus help elucidate the causal contribution of the ATL in neurologically healthy individuals. Precursor studies with young adults showed that anodal tDCS stimulation over the right ATL significantly improved retrieval accuracy (relative to the sham condition) for famous people's names but not for famous landmarks. The authors suggested that anodal tDCS modulated access to person-specific semantic information such as a person's name^13^. However, the recall of famous people's names in older adults was improved only after anodal tDCS stimulation of the left ATL (relative to the sham condition). This result does not contradict the finding for young adults as it was attributed to the decrease in lateralization that characterized the aging brain^33^. In another study involving younger adults, anodal tDCS stimulation of the left ATL impaired recall and recognition of unfamiliar faces compared to sham^34^. Possible reasons for this diversity of results might be differences in the stimuli used (familiar or unfamiliar faces) and small sample sizes (e.g., 12 participants in Pisoni et al. (2015) and 6 participants in Gorno-Tempini et al. (1998)). The interpretability of the results is also often limited by the absence of a suitable control condition (recall of memory for other terms), as the stimulation of left hemispheric areas might affect language processing in general.

In the present study, we further explored the causal contribution of the left (Experiment 1) and right (Experiment 2) ATL for proper name recall using tDCS by adding a control condition. Participants had to memorize unknown faces with information about the person's surname and profession. We also used German surnames that denoted a profession (e.g., "Herr Gärtner", *Mr. Gardener*). After tDCS stimulation of the left and right ATL, the participants were presented with faces and asked to recall name and profession. Based on the assumption that the left ATL mediates the associations between names and person-related semantic information through lexical access in the language dominant hemisphere^1,13,33,34^, we hypothesized that anodal and cathodal tDCS of the left ATL would improve and impair surname recall, respectively. For the right ATL, the neurocognitive processes underlying proper name recall are less clear. Nonetheless, based on the evidence from patient studies of its role in mediating the association between faces and person-related semantic information^10,20,29^, we hypothesized that anodal and cathodal tDCS of the right ATL would improve and impair profession recall (relative to surname recall), respectively.

## Experiment 1

### Participants

A priori power calculations indicated that for an experiment with a repeated measures within-subject design, a minimum sample size of 12 is sufficient to achieve a statistical power (1-β) of 95% at an alpha level of 0.05 and a moderate effect size (0.50) (G*Power 3.1.9.2). We recruited twenty-seven healthy volunteers (8 males, mean age: 23.18, SD: + 4.58 years) for Experiment 1. We recruited more participants than necessary to cope with potential dropouts because of the three sessions required for the experiment. Luckily, no participants were lost. All were native German speakers, had normal or corrected to normal vision and were right-handed according to the Edinburgh Handedness Inventory^35^. The participants were prescreened for contraindications to tDCS, such as metallic or electrical implants in the body or the head^36^. Volunteers with a history of chronic medical or neuropsychiatric disorders (e.g., depression, epilepsy, and stroke), learning disability, brain injuries, taking maintenance medications, and illicit drug use were excluded. The participants gave written informed consent after a detailed explanation of the experimental procedures. They were asked to avoid alcoholic drinks 24 h before the experiment, as well as caffeine and nicotine consumption at least 3 h before the experiment. Psychology students could receive course credit for the entire duration of the examination. The study was approved by the Ethics Committee of the University of Graz (reference number: 39/8/63 ex 2017/18), and all experimental procedures conformed to the guidelines set by the Declaration of Helsinki.

### Transcranial direct current stimulator (tDCS)

For tDCS, a 2 mA current was delivered via rectangular saline-soaked surface sponge electrodes connected to a battery‐driven, constant‐current DC‐stimulator (ELDITH DC-stimulator, NeuroConn, Germany). The surface area of the electrodes measured 35 cm^2^ with a current density of approximately 0.057 mA/cm^2^. The stimulating electrode was placed over the T3 EEG electrode location (International 10–20 EEG System), which corresponds to the left ATL. The reference electrode was positioned over the supraorbital area contralateral to the stimulating electrode. Therefore, we had a T3-right supraorbital area (T3-RO) montage for Experiment 1. The current was delivered for 20 min for the real stimulation conditions and slowly ramped up and down for 10 s at the start and end of the stimulation. The impedance during stimulation was maintained below 10 kΩ in order to minimize a tingling skin sensation. The same amount of current was applied in the sham stimulation condition but only for 30 s and then turned off automatically. This ensured effective blinding with regard to the stimulation conditions because the participants experienced a similar skin sensation during sham stimulation.

### Proper name retrieval task

**Figure 1:**
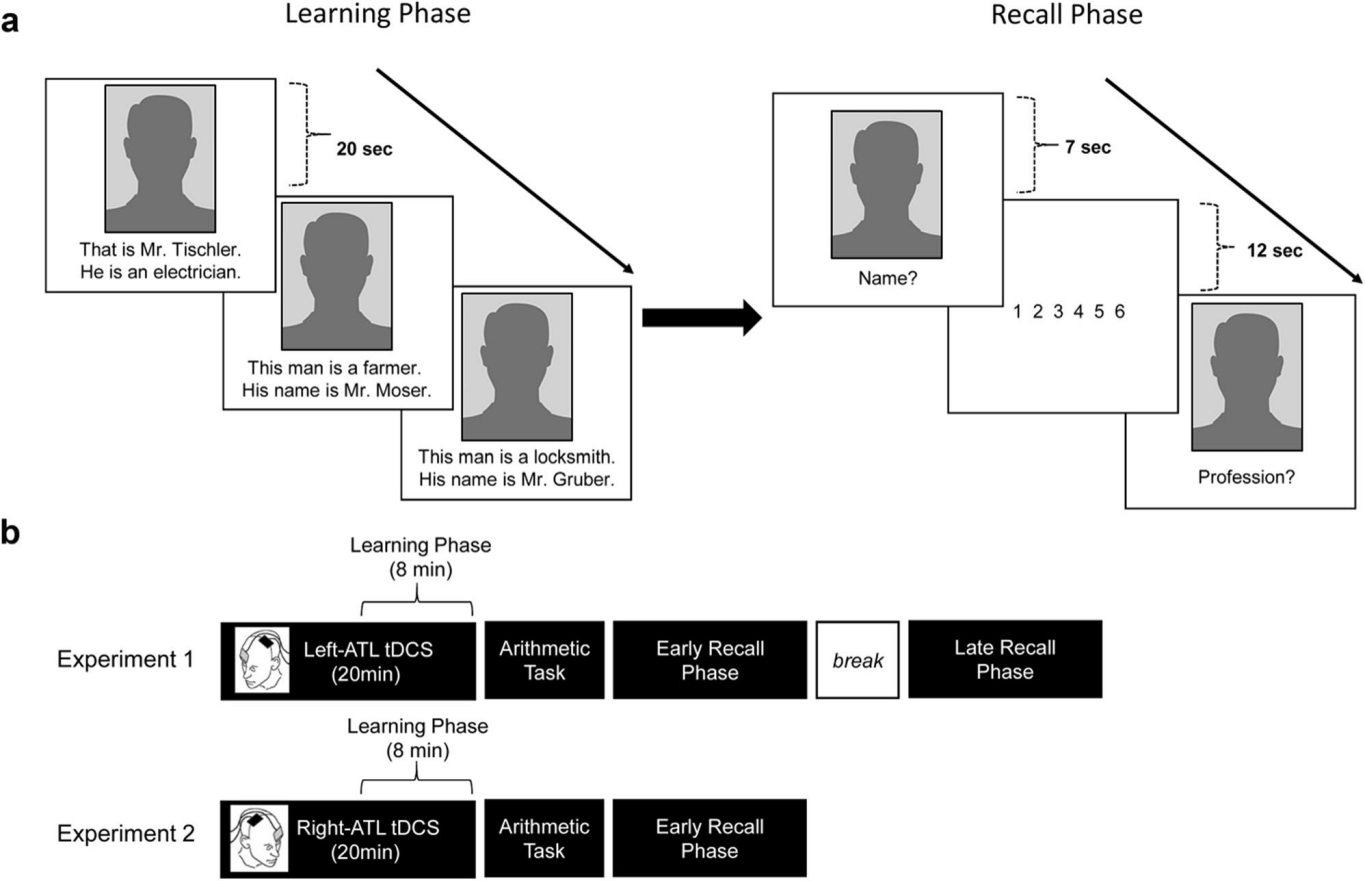
Experimental procedures (**a**) Task design. During the learning phase, the pictures and associated surname-profession pairs were presented twice for 20 s. In the recall phases, the pictures were randomly presented without the surname-profession pairs. Participants had to recall either the name or the profession assigned to the picture. A six-point confidence rating scale followed each picture. (**b**) Time course of the experiments. In Experiment 1, participants underwent three sessions of tDCS stimulation of the left ATL for 20 min. The learning phase coincided with the last 8 min of the stimulation. A one-minute arithmetic task followed the learning phase. Then the participants were asked to recall the surnames or professions immediately after the arithmetic task (early recall phase) and after a 30-min break (late recall phase). The procedure was similar for Experiment 2 except that the right ATL was stimulated, and only the early recall phase was implemented. The image of a human silhouette was adapted from Pixabay (https://pixabay.com/). tDCS = transcranial direct current stimulation, left-ATL = left anterior temporal lobe, right-ATL = right anterior temporal lobe.

The task stimuli were 24 pictures of unknown Caucasian male faces (frontal view) with a friendly smiling expression downloaded from the Karolinska Directed Emotional Faces (KDEF) database (Image IDs: AM02HAS, AM05HAS, AM06HAS, AM07HAS, AM08HAS, AM09HAS, AM010HAS, AM12HAS, AM13HAS, AM15HAS, AM17HAS, AM21HAS, AM22HAS, AM23HAS, AM24HAS, AM29HAS, AM30HAS, AM31HAS, BM04HAS, BM11HAS, BM25HAS, BM26HAS, BM27HAS and BM28HAS) (https://www.kdef.se/). ^37^. The grayscale pictures measured 15.5 cm × 21 cm on the screen, corresponding to 564 × 765 pixels (Fig. 1). We only used pictures of male faces and male profession labels because female profession labels are gender marked in German and do not exist as surnames. The pictures were divided into 3 sets of 8 pictures each. Each set was presented per experimental session. The number of pictures in a set had to be small to prevent extraneous cognitive load on the memory of the participants. For each set, 4 of the pictures were assigned surnames that were synonymous with professions (e.g., "Pfleger", nurse). These names were paired with professions that do not exist as a surname (e.g., "Maschinist", *operator*). The other 4 pictures were assigned with the opposite pairs: surnames that do not refer to a profession (e.g., "Gruber" ) together with professions that are used as a surname (e.g., "Maurer", *mason*). Typical Austrian surnames were taken from a list of landline and mobile phone subscribers (https://www.telefonabc.at), while professions were taken from an online German dictionary (www.duden.de). Easy to remember surnames such as those from popular Austrian politicians (e.g., Hofer, Berger, Baumgartner), adjectives (e.g., "Lang", long), and animal names (e.g., "Wolf", wolf) were excluded. The participants were presented with the pictures during the learning and recall phase (Fig. 1a).

In the learning phase, the participants were instructed to memorize the surname-profession pairs assigned to the presented pictures. The pictures appeared in the middle of the computer screen with the surname-profession pairs written below it. In half of the pictures, the surnames were indicated first followed by profession (e.g., "Das ist Herr Tischler. Er ist Elektriker", *This is Mr. Carpenter. He is an electrician*) and in reversed order (e.g., "Dieser Mann ist Bauer. Sein Name ist Herr Moser", *This man is a farmer. His name is Mr. Moser*) on the other half. Each picture was presented twice for 20 s each (total duration = approx. 5–6 min.). The picture presentation order across participants was randomized. After a 30 min break, the participants went through a recall phase where the same pictures were again presented, albeit in a different order. In the recall phase, only the pictures were shown (8 pictures were presented in two blocks). In the first block (8 trials), participants were instructed to recall the surnames and the professions in the second block. The order of the two blocks was counterbalanced over participants. The participants were asked to recall the requested information associated with the picture as quickly and accurately as possible. They answered into a headset microphone. The sound was recorded with the onset of the picture presentation. Picture presentation and response recording were controlled using a program based on Psychopy (Psychology Software in Python, University of Nottingham)^38^. The participant's reaction times (RTs) and errors were later analyzed from the audio files.

### Experimental design and procedure

In Experiment 1, a single-blinded, randomized, sham-controlled design was adopted. Each participant took part in three randomized stimulation sessions (two real tDCS and one sham stimulation session) separated by an interval of at least 1 week to avoid carry-over effects. The experiments were performed inside a well-lit and sound-attenuated room. Participants sat in a comfortable chair with head and arm supports in front of a 22-inch computer monitor used to present the task. The experiment began with head measurements to individually determine the left ATL location (electrode position T3 in the international 10–20 EEG system). First, we measured the distance between nasion and inion along the midplane using a tape measure. The halfway point of this distance was marked on the participant's scalp using a washable colored pencil. Second, we measured the pre-auricular distance that passes through the marked spot. The point of intersection was then designated as the vertex. EEG caps of various sizes were used in order to cover different head sizes and shapes. The EEG cap was placed on the participant's head and adjusted so that the Cz electrode was directly located on top of the vertex. The location of the T3 electrode was determined and marked on the scalp. The stimulating electrode was securely placed on the marked T3 spot and the reference electrode above the RO with an elastic bandage. Subsequently, the participants received 20-min tDCS stimulation of the left ATL. They were asked to relax and keep their eyes open during the first 12 min of the stimulation. Then an instruction appeared in the center of the screen informing the participants that pictures would be presented and that they had to memorize the surname-profession pairs presented with them. This period was the learning phase and coincided with the last 8 min of the stimulation (Fig. 1b). The learning phase finished shortly before the stimulation ended.

The tDCS electrodes were immediately removed, and the participants were given a one-minute simple arithmetic task to prevent them from using their working memory skills in the subsequent recall phase. The participants solved one-digit multiplication problems (e.g., 2 × 3), as well as one- and two-digit additions (e.g., 3 + 67) and subtraction (e.g., 87–34) problems presented on the computer screen. The operands in both operations never exceeded 100. Participants were given 6 s to type in their answer using the computer keyboard. After the arithmetic task, the first (early) recall phase followed, where the same pictures were presented without the written surname-profession pairs below. The picture presentation was randomized for each participant. Participants were asked to identify first either the surnames or professions associated with the pictures. In total, the early recall phase contained 16 trials. For each trial, the participants were given 7 s to produce the answer orally. After 7 s, the picture disappeared, and a six-point confidence rating scale appeared on the screen. Using a computer mouse, the participants ticked the number on the scale (from left to right) that corresponds to how confident they were (1—very uncertain, 2—uncertain, 3—rather uncertain, 4—more secure, 5—confident, 6—very confident). The participants were given a maximum of 12 s to rate each answer. Ticking a number activated the appearance of the next picture. The early recall phase, including the confidence rating, lasted for approximately 5 min. A 30-min break followed the early recall phase, during which the participants watched a video of a popular American sitcom. After the break, the participants underwent another (late) recall phase. In total, one experimental session, including the preparations, took about 70 min.

At the end of the experimental session, stimulation-related adverse symptoms were documented using a standard tDCS questionnaire. In addition, each participant was asked, “what type of stimulation do you think you received today, sham or real stimulation?”. The participants tolerated tDCS stimulation well, and there were no reports of any adverse effects like skin irritation, headache, and dizziness. All participants reported that they experienced the same sensation (tingling) in the target area. In the sham session, 22 participants thought they received real stimulation, 5 answered “I don't know” or “I am not sure”, and none thought they received sham stimulation. In both anodal and cathodal sessions, all participants thought that they received real stimulation. Collectively, these results suggest that the blinding procedure was successful.

### Statistical analysis

To explore the effect of the stimulation on proper name recall, the experimenter determined the participants' RTs and errors from individual audio files using the software Audacity for Windows (www.audacityteam.org/). The RT (in seconds) was defined as the period between the picture presentation and the beginning of the participant's utterance of the surname or profession. RTs from incorrect trials and trials with RTs outside of + 2 SD of the general mean (outliers) were excluded from the data. Pearson's correlation coefficient was computed to determine the correlation between the RTs from correct trials and corresponding confidence ratings. The participant's error rates (ERs%), on the other hand, were computed by the formula incorrect trials/total number of trials × 100. ERs were separately calculated for each error category: "intrusion" when the participants provided a wrong surname or profession and "omission" when participants failed to produce a reaction within the given time limit^34^. Before data analysis, we first verified the distribution of the dependent measures and homogeneity of variance using Shapiro–Wilk and Levene's test, respectively. Violation of normal data distribution and homogeneity of variance were dealt with using log-transformation of the data. The participant's ERs and trial-by-trial RTs were modelled separately using linear mixed-effects modelling (LMM) with random-intercept in SPSS 26 software (IBM SPSS Statistics, IBM Corp., Armonk, NY, USA). For our data, LMM is a robust alternative to pure ANOVAs because we had an unequal number of observations per participant (after discarding outliers and wrong trials) on each experiment^39,40^. Additionally, LMM allows greater control of between-subjects variability and can incorporate participant-specific characteristics into the model^41^. For the analysis of the RTs (log-transformed), the model in Experiment 1 contained the within-subjects factor stimulation (anodal, cathodal and sham), person-specific information (surnames, professions), and time of recall (early, late) as fixed-effect covariates. For the analysis of the ERs, the model contained one additional fixed factor, error type (intrusions, omissions). A participant-specific intercept was included in the models as a random-effect covariate.

To test the adequacy of the model fit on the data, we performed model comparison procedures. Here, we conducted a forward stepwise approach by adding the fixed-effect covariates (within-subjects factors followed by the between-subjects factor, and their respective interactions) one at a time to a baseline model that only contained the random-effect factor participants^42^. The maximum likelihood (ML) estimation (Compound Symmetry model) was used to fit all models' mixed-effects. The Akaike Information Criterion (AIC) value of the initial model and the next model were compared. A decrease and increase of AIC value by a factor of 2 indicate improving and worsening model fit, respectively^43,44^. However, an AIC value only compares one model to the next and does not indicate the model's absolute fit to the data; therefore, we also calculated the Akaike weight of each model^44^. The Akaike weights can be used to compare all possible models and determine which model will come out best most of the time. In the final model, collinearity was tested by determining the tolerance and variance inflation factors. Factors with nonsignificant main effects were excluded except when they were involved in significant higher interactions. Significant findings from the models were explored using post hoc comparisons (paired t-test, two-tailed, Bonferroni adjusted for multiple comparisons). We calculated Cohen’s *d* as a measure of effect size (< 0.2—trivial, > 0.2—small, > 0.5—medium and > 0.8—large). A *p*-value of < 0.05 was considered significant for all statistical analyses. All values are expressed as the mean ± standard error of the mean (SEM).

### Results

All data were log-transformed to ensure normal data distribution (Shapiro–Wilk test) and to keep the variances equal (Levene's test) (all *p* > 0.05). In the final model, tolerance and variance inflation factors were equal to 1.000, indicating that multicollinearity did not affect the findings.

#### RTs

**Figure 2:**
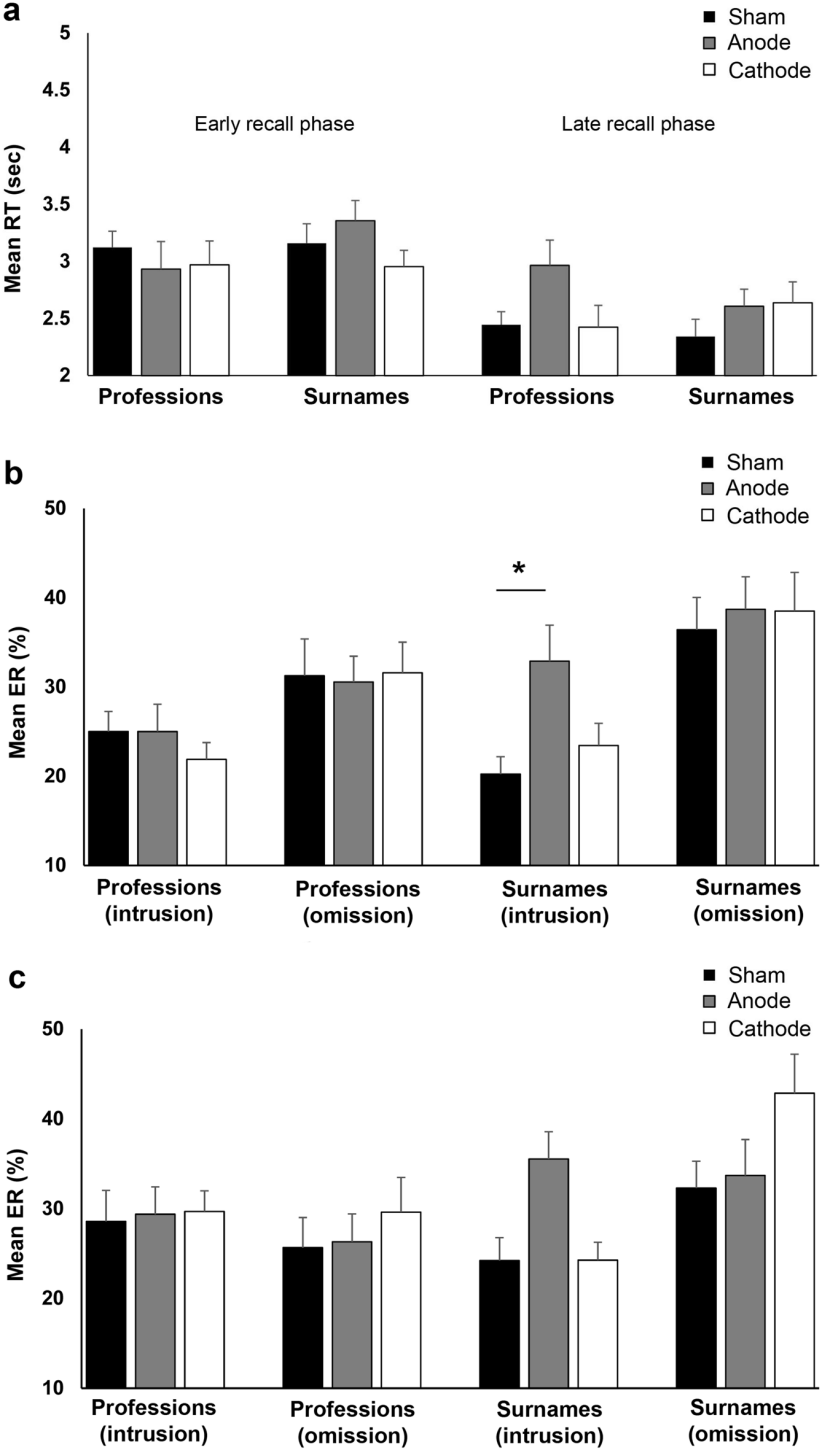
Effects of left ATL tDCS on the recall of person-specific information (**a**) Reaction times (RT) during the early and late recall phases. The x-axis displays the person-specific information and stimulation conditions in the early and late recall phases. The y-axis represents the mean RT (sec) for correct trials. Overall, RTs were significantly shorter for professions than surnames and the late recall phase than the early recall phase. (**b**) Early and (**c**) late recall phase’ error rates (ERs). The x-axis displays the person-specific information and error type per stimulation conditions. The y-axis represents the mean ERs (%). Overall, intrusions are significantly higher in the anodal condition compared to sham and cathodal conditions. *Indicates significant differences in ERs between stimulation conditions. Error bars represent the standard error of mean (SEM).

RTs from incorrect trials and those classified as outliers accounted for 25.56% (464) and 0.33% (6) of the whole dataset (1815), respectively. They were discarded and not included in the final model. Therefore, the results from Experiment 1 were based on the remaining 74.1% (1354 trials) of the data. The initial model containing all the factors satisfied the goodness-of-fit of the data based on the AIC values, and a model containing the factor time will come out best 34% of the time based on the Akaike weights (Supplementary Table S1). However, the results of the full model revealed that tDCS had not impacted the RT, as indicated by the nonsignificant main and interaction effect of the factor stimulation (Supplementary Table S2). The main effect of person-specific information and time of recall were significant. Therefore, we decided to run and interpret a reduced model containing these factors and their interactions (Table 1). The result of the reduced model was consistent with the Baker-baker paradox: professions (M = 2.67 s, SD = 0.39 s) were easier to recall than surnames (M = 2.82 s, SD = 0.42 s), which led to a significant main effect of person-specific information (Table 1, Fig. 2a). The main effect of the factor time of recall was also significant as indicated by the shorter RTs in the late recall phase (*M* = 2.55 s, *SD* = 0.40) than in the early recall phase (*M* = 2.94 s, *SD* = 0.42) (Fig. 2a). However, RTs for professions and surnames did not significantly differ across the two recall phases, as shown by the nonsignificant interaction of person-specific information and time of recall. The confidence ratings correlated negatively with the RTs, indicating that faster retrieval was associated with higher confidence (professions: early recall: r = − 0.44, n = 368, *p* =  < 0.001; late recall: r = − 0.66, n = 389, *p* =  < 0.001; names: early recall: r = − 0.54, n = 284, *p* =  < 0.001; late recall: r = − 0.51, n = 309, *p* =  < 0.001).

**Table 1:** Results of the linear mixed (reduced) model (LMM) performed on the reaction time (RT) and error rate (ER) in Experiment 1.

	Numerator df	Denominator df	*F*-value	*p-*value	Cohen's d
**RT**					
Person-specific information	1	1330.10	6.27	.012*	.452
Time of recall	1	1322.15	36.03	< .001*	.985
Person-specific information × time of recall	1	1322.48	.77	.380	.208
**ER**					
Stimulation	2	452.61	2.62	.074	.309
Person-specific information	1	452.47	9.21	.003*	.557
Error type	1	450.47	17.35	< .001*	.765
Stimulation × person-specific information	2	449.80	1.51	.223	.248
Error type × person-specific information	1	446.65	7.81	.005*	.461
Stimulation × error type	2	447.60	3.48	.032*	.553
Person-specific information × stimulation x error type	2	449.17	1.33	.265	.270

In the models, each participant was treated as a random factor (random intercept model). The within-subjects factors stimulation (anodal, cathodal and sham), person-specific information (surnames, professions), and time of recall (early, late) were treated as fixed-effect covariates for the RT model. For the ER model, the factor time of recall was replaced by error type (intrusions, omissions). Asterisks indicate significant results (*p* < 0.05). df, Degrees of freedom.

#### ERs

The results of the full model for the ERs revealed a nonsignificant main effect of the factor time, indicating a comparable total number of errors in the early (51.9%) and late (48.1%) recall phases (Supplementary Table S2). The three- and four-way interactions involving the factor time were also not significant; therefore, we ran and interpreted a reduced model without this factor (Table 1). The results of the reduced model are again consistent with the Baker-baker paradox. Overall, the ER was significantly higher for surnames (53.6%) than professions (46.4%), resulting in a significant main effect of the factor person-specific information (Fig. 2b,c). Participants committed more omissions (52.3%) than intrusions (47.7%) errors, as indicated by the error type's significant main effect. However, Bonferroni-corrected post-hoc *t*-tests showed that this difference was more pronounced for surnames (omissions: M = 36.37%, SD = 9.13%, intrusions: M = 26.17%, SD = 9.68%, *p* < 0.001) than for professions (omissions: M = 27.83%, SD = 9.95%, intrusions: M = 25.79%, SD = 9.87%, *p* < 0.861). The stimulation affected error rates, but this effect depended on the error type indicated by the significant interaction of stimulation and error type (Table 1). Intrusions were significantly more numerous in the anodal condition compared to sham (*p* = 0.027) and cathodal condition (*p* = 0.043), while intrusions between sham and cathodal conditions were comparable (*p* = 0.999). On the other hand, the number of omissions did not significantly differ between sham and anodal stimulation (*p* = 0.999), between sham and cathodal stimulation (*p* = 0.218), and between anodal and cathodal stimulation (*p* = 0.653) (Fig. 2b,c). Exploratory post hoc comparison revealed that intrusions were higher for surnames in the anodal condition compared to sham in the early (*p* = 0.019) but not late (*p* = 0.082) recall phase (Fig. 2b,c).

### Discussion of experiment 1

In Experiment 1, we replicated the Baker-baker paradox since participants were faster and more accurate in recalling professions than surnames. We also observed more omission errors than intrusions, especially for surnames. This indicates that participants had more difficulties with surname recall than with profession recall. The difference between the error types with regard to surname and profession recall also indicates that the participants encountered greater difficulty retrieving the surname from memory. Concerning the stimulation-specific effect, we did not observe significant changes in reaction times, but we observed a polarity-dependent effect on the intrusion errors for surnames. Anodal stimulation significantly increased the intrusion errors for surnames compared to sham stimulation in the early but not late recall phase. This result suggests the crucial role of the left ATL in the recall of proper names.

Pisoni and colleagues also observed increased intrusion errors after anodal stimulation of the left ATL when their participants recalled names^34^. It is possible that anodal tDCS increased neuronal excitability, which added noise to the neural network involved in recalling proper names and professions. Suppose our hypothesis is correct that person-specific information is stored in the right ATL, then perturbations in the left ATL should not interfere with surname recall. This seems to be the case because anodal tDCS interfered with surname but not profession recall. Considering the functional model of face recognition, we can, in theory, argue that participants were able to access the FRU and access semantic information from PINs allowing them to identify their professions. However, they failed on the final step (naming), which is access to the lexical unit corresponding to the person’s surname (access to name code)^5,10,11^. However, the higher number of intrusion errors (articulation of wrong surnames or saying a profession instead of surnames) after anodal stimulation cannot be easily explained by the failure to access name codes that are supposed to elicit more omission errors (complete retrieval failure). In this case, we can adapt the interactive activation and competition (IAC) models that focus more specifically on the linguistic processes^5,10^. Based on these models, semantic information is not contained in PINs and is only accessible through tokens made within the PINs. Since activations of semantic and lexical information by tokens occur in parallel, interference from left ATL anodal tDCS could, in theory, affect surnames more than professions because of the preponderance of the left hemisphere in this stage^10^. Specifically, the interference might have been caused by the co-activation of competing lexical information (e.g., surnames associated with other faces) or confusion whether a recalled word was a surname or a profession boosted by anodal stimulation.

Interestingly, one study reported increased proper name retrieval accuracy after anodal tDCS of the left ATL (relative to sham)^33^. In that study, elderly adults were tested on the naming of famous faces. This is different from the present experiment that used younger adults and learned names and professions to unknown faces. Retrieval of person-specific information from faces of an unknown and famous person differs because of the facial distinctiveness effect^45,46^, and therefore can be modulated differently by anodal stimulation. In theory, there will be less neuronal noise (few competing faces or names) when retrieving a famous persons’ name because the stimulus (a familiar face) is distinct. In contrast, a higher noise level (more competing surnames) is expected when retrieving unknown individuals' surnames because the stimuli (unfamiliar faces) are less distinct. Unfamiliar faces have no semantic information compared to familiar/ famous faces that link to biographic information^12^. Since access to name codes is assumed to operate via a single-route process (PINs to name code) according to the face recognition model^5,11^, recall of proper names for unfamiliar faces would be more susceptible to interference than for familiar faces. This may explain why the effect of anodal tDCS is detrimental to our task but facilitatory to the task in the study of Ross et al. (2011).

For cathodal tDCS, similar to the result of the Pisoni et al. study (2015), recall of person-specific information is neither impaired nor enhanced by the stimulation. Here, we argue that left ATL's cathodal stimulation might not have been sufficient to inhibit the recall of person-specific information. This is because other brain regions besides the left ATL are reported to have a complementary function in recalling person-specific information^1^. For instance, processes that may aid recall, such as access to lexical and phonological information, are a function of the left inferior frontal gyrus (IFG)^10,47,48^. Indeed, probably due to interference, anodal stimulation of the left IFG also elicits errors in recalling proper names^34^. The dorsolateral prefrontal cortex, posterior cingulate cortex, and angular gyrus were also associated with the retrieval of person-specific semantic information^10,25^. The absence of an inhibitory effect on profession recall also fits our original hypothesis that the right ATL might be a brain region mediating the association between faces and person-related semantic information^1,34^. To test this hypothesis, we performed right ATL tDCS in the second experiment.

## Experiment 2

### Participants

For the second experiment, twenty-seven volunteers were also recruited. However, due to a technical problem on the recording device, the data of seven participants were not saved; hence only the data from twenty young, healthy participants (13 males, mean age: 24.45, SD: ± 4.09 years) were used in the analysis for Experiment 2. None of them participated in Experiment 1. The same screening criteria and procedures were implemented in Experiment 2. The participant's demographic characteristics (university students, native German speakers, had normal or corrected to normal vision and were right-handed) were comparable to those in Experiment 1. Psychology students received course credit equivalent to the time they spent in the second experiment.

### Transcranial direct current stimulator (tDCS)

The tDCS procedures and parameters were identical to Experiment 1 except for the electrode montage. For Experiment 2, the stimulating electrode was placed over the T4 EEG electrode location (International 10–20 EEG System) which corresponds to the right ATL, and the reference electrode was positioned over the left supraorbital area (LO) (Fig. 1).

### Proper name retrieval task and experimental procedure

The experimental design and procedures for Experiment 2 were identical to that of Experiment 1 (Fig. 1). The tDCS electrodes with T4-LO montage were securely placed on the head with an elastic bandage. Using the same picture-surname-profession pairs from Experiment 1, participants performed the learning phase in the last 8 min of the 20-min stimulation period. The one-minute simple arithmetic task followed the learning phase. For the recall phase, we introduced some modifications (Fig. 1b). First, we removed the late recall phase because, in Experiment 1, the effects of the stimulation had been the same for both phases. Second, to ensure that the allotted time to respond was sufficient, participants in Experiment 2 were given 15 s to respond. Therefore, Experiment 2 only took on average 35 min to finish. The participants in Experiment 2 tolerated tDCS stimulation well, and there were no reports of adverse effects. Concerning the blinding, no participant thought receiving sham stimulation during the sham session (18 thought receiving real stimulation and 2 were undecided (answered “I am not sure”)). Meanwhile, all participants thought they received real stimulation during the anodal and cathodal sessions. This indicates that the blinding procedure was largely successful.

### Statistical analysis

The same statistical procedure, including model selection, was performed for the RTs and ERs in Experiment 2. The final model for the RTs contained the within-subjects factor stimulation (anodal, cathodal and sham) and person-specific information (surnames, professions) as fixed-effect covariates. There was no within-subject factor time of recall since there was no late recall phase in Experiment 2. Pearson's correlation coefficient was also determined to explore the correlation between the RTs from correct trials and corresponding confidence ratings. Similar to Experiment 1, the model for the ERs contained the within-subjects factors stimulation (anodal, cathodal and sham) and person-specific information (surnames, professions), but had an additional within-subject factor error type (intrusion, omission).

### Results

Log-transformed data were normally distributed (Shapiro–Wilk test), and the variances equal (Levene's test) (all *p* > 0.05). Multicollinearity was also unproblematic in the final models in Experiment 2 since the tolerance and variance inflation factors were equal to 1.000.

#### RTs

**Figure 3:**
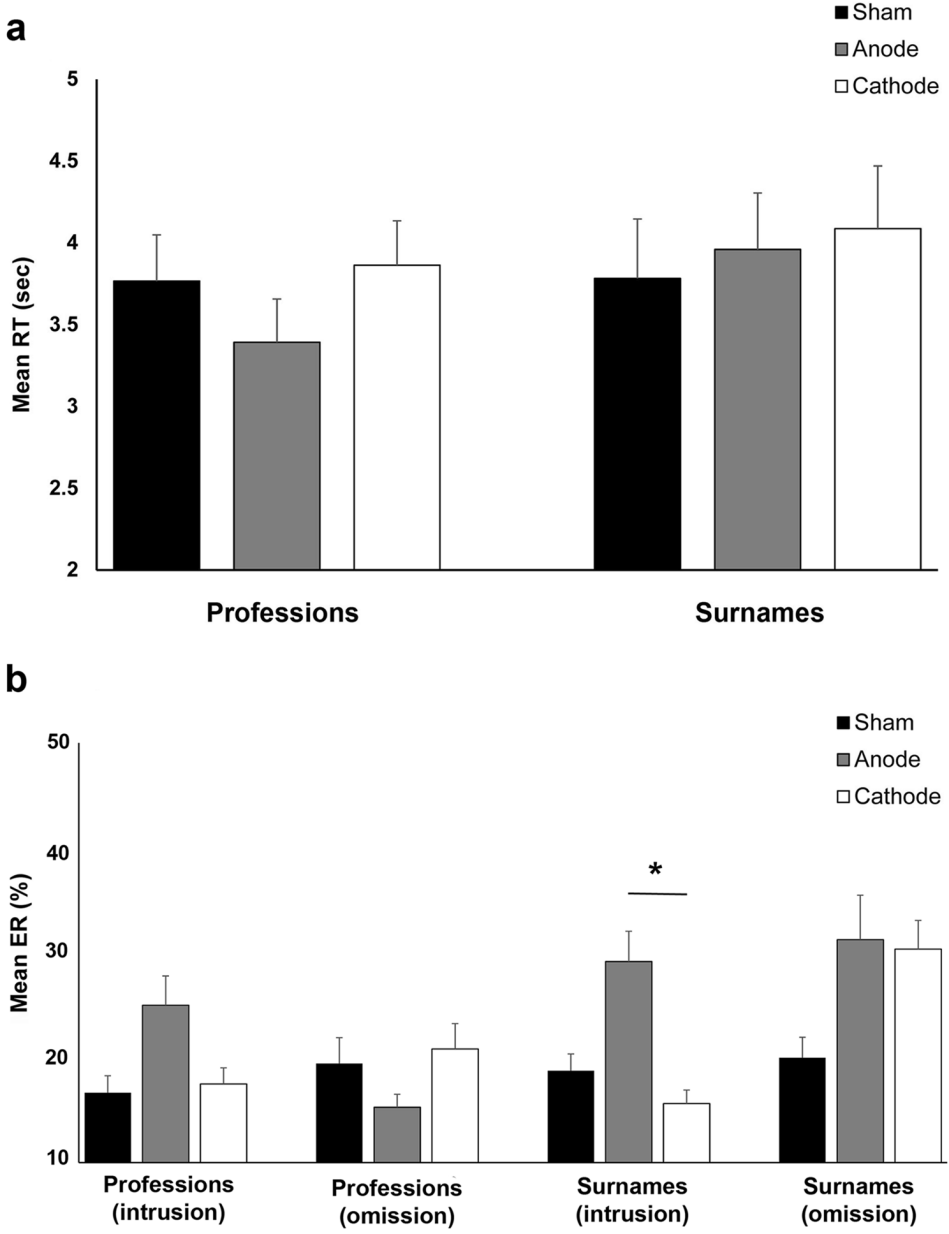
Effects of right ATL tDCS on the recall of person-specific information (**a**) Reaction times (RTs) during early recall. The x-axis displays the person-specific information and stimulation conditions. The y-axis represents the mean RTs (sec) of correct trials. Overall, RTs were significantly shorter in anodal condition compared to cathodal condition. (**b**) Error rates (ERs) during early recall. The x-axis displays the error type, person-specific information and stimulation conditions. The y-axis represents the mean ERs (%). Overall, there were more intrusions in the anodal condition compared to the cathodal condition. * Indicates significant differences in ER between stimulation conditions. Error bars represent the standard error of the mean (SEM).

In Experiment 2, there were 202 incorrect trials (21.09%) and 67 data points that were classified as outliers (6.99%) from a total of 958. They were discarded, and the analysis was conducted on the remaining 71.92% (689 trials) of the data. We interpret a model without the factor error type since it had no significant main and interaction effect in the initial analysis. In addition, based on the Akaike weights, the final model containing the factor stimulation and a model containing the stimulation and person-specific information will come out the best 35% and 27% of the time, respectively (Supplementary Table S1). The results showed that the stimulation modulated RTs as indicated by the significant main effect (Table 2). The overall RT was significantly shorter after anodal stimulation (M = 3.60 s, SD = 0.22 s) than after cathodal stimulation (M = 4.01 s, SD = 0.25 s, *p* = 0.015, Bonferroni-corrected post-hoc *t*-test, Fig. 3a). Meanwhile, overall RT after sham stimulation (M = 3.84 s, SD = 0.22 s) did not significantly differ to RTs after anodal (*p* = 0.560) and cathodal (*p* = 0.438) stimulation. Similar to Experiment 1, confidence ratings were higher for faster reaction time (professions: r = − 0.61, n = 135, *p* =  < 0.001; surnames: r = − 0.51, n = 309, *p* =  < 0.001).

**Table 2:** Results of the linear mixed model (LMM) performed on the reaction times (RTs) and error rates (ERs) in Experiment 2.

	Numerator df	Denominator df	*F*-value	*p*-value	Cohen's *d*
**RT**					
Stimulation	2	717.85	3.98	.019*	.350
Person-specific information	1	718.17	1.12	.290	.169
Stimulation × person-specific information	2	716.36	.52	.595	.137
**ER**					
Stimulation	2	75.23	2.68	.075	.447
Person-specific information	1	71.24	4.19	.044*	.552
Error type	1	73.70	1.06	.308	.279
Stimulation × person-specific information	2	69.24	.49	.613	.323
Stimulation × error type	2	72.56	3.73	.029*	.456
Person-specific information x error type	1	70.52	2.54	.115	.339
Stimulation × person-specific information × error type	2	71.64	.88	.418	.368

In the model, each participant was treated as a random factor (random intercept model). The within-subjects factors stimulation (anodal, cathodal and sham) and person-specific information (surnames, professions) were treated as fixed-effect covariates for the RT model. The within-subject factor error type was added to the model of the ER. Asterisks indicate significant results (*p* < 0.05). df, Degrees of freedom.

#### ERs

The stimulation and error type influenced the error rates, which is why we interpret a full model (Table 2). The overall number of errors for surnames (M = 23.51%, SD = 7.66%) was significantly higher than errors for professions (M = 18.59%, SD = 8.38%, Fig. 3b), which is reflected in a significant main effect of error type. The stimulation's effect depended on the type of error, as indicated by the significant stimulation and error type interactions (Table 2). There were more intrusions in the anodal condition (26.81%) than in the cathodal condition (16.36%, *p* = 0.048, Bonferroni-corrected post-hoc *t*-test). Intrusions between sham and cathodal conditions (*p* = 0.999), as well as between sham and anodal condition (*p* = 0.054) were comparable ( Bonferroni-corrected post-hoc *t*-test). Exploratory post hoc comparisons also revealed that intrusions were significantly higher only for surnames in the anodal condition than the cathodal condition (*p* = 0.033) (Fig. 3b). Intrusions for surnames were comparable between sham and anodal condition (*p* = 0.179), as well as between sham and cathodal condition (*p* = 0.999). There were no significant differences in the number of omission errors between stimulation conditions (all *p*s =  > 0.050).

### Discussion of experiment 2

In Experiment 2, the Baker-baker paradox was also evident because participants were more accurate in recalling professions than surnames. Anodal stimulation of the right ATL significantly accelerated overall RTs compared to cathodal stimulation. For the ERs, anodal stimulation increased the number of intrusions (relative to omissions) compared to cathodal stimulation. Interestingly, the exploratory comparisons revealed that the increase in intrusions after anodal stimulation was more robust for surnames (Fig. 3b). We want to stress that the effects of anodal tDCS on both RTs and ER are insignificant compared to sham. Nonetheless, we believe they merit discussion because they are specific for anodal tDCS and may indicate genuine retrieval interference due to the noise induced in the neural network. If this was the case, the effect, particularly on the ERs was unexpected because we hypothesized that tDCS would only affect profession recall since the right ATL was proposed to mediate the association between faces and person-related semantic information^1,34^. Arguing on the basis of the functional model of face recognition^5,11^, anodal stimulation may have interfered with a sense of familiarity (e.g., “this is a young white man that I saw before”), which is associated with the right ATL^30^. Since face recognition or familiarity judgement is the initial step towards face naming, once it is interrupted, downstream processes such as access to PINs and subsequent access to the name is no longer possible. In other words, interference with visual familiarity in right ATL may not be compensated by an intact semantic function of the left ATL. This scenario is reminiscent of patients with the semantic variant of primary progressive aphasia, those with right-predominant ATL atrophy exhibit a deficit in all tasks (e.g., familiarity judgments, semantic association, and naming of famous faces), whereas those with left-predominant ATL atrophy exhibit poor scores in semantic/biographical knowledge and naming but with spared feelings of familiarity, especially for personally known faces^30^.

Faster RTs (relative to sham) for naming famous faces after anodal stimulation of the right ATL were also reported in young adults by another study^13^. However, although Ross and colleagues reported an increased accuracy for longer RTs (> 5secs), the average accuracy for name recall was similar across stimulation conditions. For older adults, anodal stimulation of the right ATL had no significant effect on accuracy and RT for naming faces but was associated with a slowing of RT when place names were recalled^33^. The results of the previous studies are difficult to compare with our findings because they used famous faces or contrasted name recall with the recall of place names. Together, the available evidence from previous studies and the results of Experiment 2 suggest that the right ATL’s role as a repository of person-related semantic information is not supported. In our study, the effect of anodal stimulation is not robust (only significant compared to cathodal stimulation). Contrary to our hypothesis, right ATL stimulation seems to affect the recall of both surnames and person-related semantic information. Therefore, for proper name recall, the only strong evidence for the right ATL’s involvement are the faster RTs observed for famous faces after anodal stimulation in the Ross et al. (2010) study. On the other hand, similar to Experiment 1, right ATL cathodal stimulation also had no robust effect on recalling person-specific information. We argue that the inhibitory effect of cathodal tDCS on neuronal excitability in the right ATL was too weak to impair the recall of person-specific information, which is also additionally performed by the DLPFC, posterior cingulate cortex, and angular gyrus^10,25^. The retrosplenial cortex was also active during the retrieval of past autobiographical experiences, recent or remote, emotional or neutral^49^.

## General discussion

The present study aimed at exploring the causal role of the ATLs in proper name retrieval using tDCS. Experiment 1 was designed to test the hypothesis that retrieving proper names from memory such as a person's surname is a function of the left ATL because it is assumed to mediate the associations between names and person-related semantic information^1,6,30^. On the other hand, in Experiment 2, we tested the hypothesis that retrieval of other person-specific information such as professions is a function of the right ATL because it is assumed to mediate the association between face familiarity and person-related semantic information^6,13,30,33^ The results of Experiment 1 seems to support the crucial role of the left ATL in proper name recall because anodal stimulation interfered with surname retrieval. In contrast, the results of Experiment 2 appear inconclusive because anodal stimulation does not selectively interfere with profession recall. It is possible that the low number of trials might be partly responsible for this pattern of results and should be considered a potential limitation of the study. Nevertheless, the results of the two experiments do not entirely deviate from what the functional model of face recognition, which suggests that proper names (e.g., person's surname) and identity-specific semantic information (e.g., person’s profession) are stored in separate semantic memory nodes^5,11^. Access to proper names such as surnames is significantly affected by left ATL anodal stimulation because access to identity-specific semantic information (e.g., profession), a proposed function of the right ATL, is intact. On the other hand, although interference in the right ATL probably affected the initial sense of familiarity, we cannot completely rule out that intrusions in profession and surname recall during right ATL anodal stimulation are primary and secondary effects, respectively . In other words, the interference to surname recall also occurs because of the initial interference in accessing the identity-specific semantic information (surnames). This is possible because the ATLs contribute to some degree to the parallel recall of both information types from memory, probably via inter-hemispheric connections as suggested by several neuroimaging studies showing bilateral activation during access to person-specific semantic information^10,25,50^. ERP studies also showed that access to lexical information in the left hemisphere due to its linguistic nature paralleled the access to semantic information, 300 and 600 ms after face recognition^10^.

The absence of cathodal tDCS-induced inhibition in Experiment 1 and 2, as well as in the study of Pisoni et al. (2015) is puzzling. The simplest explanation would be that neuronal excitability reduction in both ATLs might not be robust enough to impair memory recall because other brain regions, including the retrosplenial cortex, DLPFC, posterior cingulate cortex, and angular gyrus, might compensate for them^10,25,49^. Another possible explanation is the shift in the direction of excitability alterations for cathodal tDCS but not anodal tDCS. In the motor cortex, a study using the same stimulation parameters (20 min duration, 2 mA intensity, 35 cm^2^ electrodes) reported increased cortical excitability after cathodal stimulation^51^. However, if that was the case in the ATLs, why are the cathodal tDCS and sham stimulation after-effects comparable. In the same vein, why did cathodal tDCS not interfere with memory recall like anodal tDCS? The study of Batsikadze et al. (2013) might offer an explanation. In their study, enhanced cortical excitability caused by cathodal tDCS was only significant at a later time (90 and 120 min after stimulation) compared to the enhanced cortical excitability after anodal tDCS. Therefore, we can theoretically argue that the effect of cathodal stimulation may not be observable in our task design. This assumption, however, must be systematically explored by future studies.

## Conclusion

The present study highlights the role of the left ATL in the recall of proper names such as a surname. However, the evidence for the role of right ATL in recalling person-specific information could not be substantiated. These findings highlight the potential of non-invasive brain stimulation techniques in exploring the neural correlates of proper name processing. These techniques can then also be used as future neurorehabilitation tools for enhancing proper name recall, particularly among older adults. Future studies with alternative experimental paradigms (e.g., bilateral ATL tDCS stimulation or unilateral stimulation with high-definition tDCS) may help us further elucidate the precise contribution of the ATLs in proper name retrieval.

## Supplementary Information

Supplementary Information.

## Data Availability

The datasets generated during and/or analysed during the current study are available from the corresponding author on reasonable request.
