# Supplementary material for: Modulation of proper name recall by transcranial direct current stimulation of the anterior temporal lobes
Source: Sci Rep. 2022 Apr 6;12:5735. doi: 10.1038/s41598-022-09781-x (PMC8987057; doi:10.1038/s41598-022-09781-x)
Supplement: Supplementary file 1 — Supplementary Information. [file 41598_2022_9781_MOESM1_ESM.pdf]

Scientific Reports

Modulation of proper name recall by transcranial direct current stimulation of the anterior temporal lobes

Shane Fresnoza[1,3], Rosa-Maria Mayer[1], Katharina Sophia Schneider[1], Monica Christova[2,4], Eugen Gallasch[2], Anja Ischebeck[1,3]

[1] Institute of Psychology, University of Graz, Graz, Austria

[2] Otto Loewi Research Center, Division of Physiology, Medical University of Graz, Graz, Austria

[3] BioTechMed, Graz, Austria

[4] Institute of Physiotherapy, University of Applied Sciences FH-JOANNEUM, Graz, Austria

Corresponding author:

Shane Fresnoza, MD PhD

University of Graz

Institute of Psychology

Universitätsplatz 2/DG

8010 - Graz (Austria)

Tel.: +43 316 380 8503

E-mail: shane.fresnoza@uni-graz.at

Supplementary Table S1. The Akaike Information Criterion (AIC) and Akaike weights of the competing models for reaction time in Experiment 1 and 2.

**Parameters**	**AIC**	**Δ_i (AIC)**	Relative model likelihoods	**w_i (AIC)**
**Experiment 1**				
Random intercept	4707.13	29.82	0.00	0.00
+ Stimulation	4709.05	31.74	0.00	0.00
+ Stimuli	4706.84	29.53	0.00	0.00
+ Time	4677.31	0	1.00	0.34
+ Stimulation x stimuli	4679.96	2.64	0.37	0.13
+ Stimulation x time	4679.35	2.04	0.28	0.09
+ Stimuli x time	4679.96	2.64	0.37	0.13
+ Stimulation x stimuli x time	4681.66	4.34	0.88	0.30
			Sum = 2.90	
**Experiment 2**				
+ Random intercept	82.25	4.59	0.88	0.31
+ Stimulation	77.66	0	1.00	0.35
+ Stimuli	78.87	1.23	0.18	0.06
+ Stimulation x stimuli	81.77	4.11	0.78	0.27
			Sum = 2.84	

Note. The differences (Δ_i (AIC)) in AIC with respect to the AIC value of the best candidate model (model with the lowest AIC value) was first calculated. From Δ_i (AIC), we determined the relative model likelihoods. The relative model likelihoods were then normalized by dividing each value to the sum of the likelihoods of all models to determine the Akaike weight (w_i (AIC)) of each model. AIC = Akaike Information Criterion value.

Supplementary Table S2. Results of the linear mixed (full) models (LMM) performed on the reaction time (RT) and error rates (ERs) in Experiment 1.

	Numerator df	Denominator df	*F*-value	*p*-value	Cohen's d
**RT**					
Stimulation	2	1345.52	1.44	.236	0.226
Person-specific information	1	1338.27	4.98	.026*	0.410
Time of recall	1	1328.09	32.24	<.001*	0.869
Stimulation x person-specific information	2	1335.13	.73	.483	0.075
Stimulation x time of recall	2	1329.69	2.31	.099	0.092

Person-specific information x time of recall	1	1328.28	1.63	.202	0.306
Stimulation x person-specific information x time of recall	2	1328.74	1.15	.317	0.172
**ER**					
Stimulation	2	452.59	2.53	.081	.301
Person-specific information	1	452.12	9.15	.003*	.548
Time of recall	1	445.47	.06	.804	.044
Error type	1	450.55	17.11	<.001*	.751
Stimulation x person-specific information	2	449.85	1.50	.223	.277
Error type x person-specific information	1	446.67	8.16	.004*	.457
Stimulation x error type	2	447.55	3.36	.036*	.335
Stimulation x time of recall	2	445.35	.57	.567	.253
Person-specific information x time of recall	1	444.51	.01	.959	.457
Error type x time of recall	1	445.61	5.04	.025*	.626
Person-specific information x stimulation x error type	2	448.99	1.36	.259	.250
Person-specific information x stimulation x time of recall	2	445.53	.18	.835	.209
Person-specific information x error type x time of recall	1	444.85	.57	.450	.398
Stimulation x error type x time of recall	2	444.38	.247	.781	.304
Person-specific information x stimulation x error type x time of recall	2	446.38	.780	.459	.202

Note. For the LMM (random intercept model), each participant was treated as a random factor. For the RTs, the within-subjects factors stimulation (anodal, cathodal and sham), person-specific information (surnames, professions), and time of recall (early, late) were treated as fixed-effect covariates. For the ERs, error type (intrusions, omissions) was added as a fixed-effect covariate. Asterisks indicate significant results ($p < 0.05$). df, Degrees of freedom.
